# Supplementary material for: The Governance of Traffic Noise Impacting Pedestrian Amenities in Melbourne Australia: A Critical Policy Review
Source: Int J Environ Res Public Health. 2024 Aug 16;21(8):1080. doi: 10.3390/ijerph21081080 (PMC11354694; doi:10.3390/ijerph21081080)
Supplement: Supplementary file 1 [file ijerph-21-01080-s001.zip › ijerph-3074386-supplementary/Table S1 PRISMA-S Checklist.pdf]

PRISMA-S Checklist

Section/topic	#	Checklist item	Location(s) Reported
**INFORMATION SOURCES AND METHODS**			
Database name	1	https://www.vic.gov.au<br>https://www.planning.vic.gov.au<br>www.infrastructure.gov.au<br>https://www.infrastructureaustralia.gov.au<br>https://www.humanrights.vic.gov.au<br>https://www.epa.vic.gov.au<br>https://www.nepc.gov.au<br>https://www.austlii.edu.au<br>https://www.legislation.gov.au<br>https://www.parliament.vic.gov.au<br>https://ncc.abcb.gov.au/<br>https://content.legislation.vic.gov.au<br>https://www.health.gov.au/resources/publications<br>https://www.vicroads.vic.gov.au	14
Multi-database searching	2	None	
Study registries	3	https://www.who.int/publications-detail-redirect/9789289002295<br>http://www.unep.org/resources/frontiers-2022-noise-blazes-and-mismatches<br>https://www.abs.gov.au/statistics/people/population/population-projections-australia/latest-release<br>https://publications.jrc.ec.europa.eu/repository/handle/JRC72550<br>https://www.iso.org<br>https://op.europa.eu/en/publication-detail/	6
Online resources and browsing	4	Database search criteria options varied slightly for each library. Search terms included: Transport AND Noise OR Health AND Noise OR Environment AND Noise + [at least one of the words]: policy, plan, provision, regulation, instrument, act, guidelines, standards, surveys, programs, strategies, frameworks, report, protocol.	

		Title and content = Council Plan, Community Plan, All types of Legislation<br>Research, standards or policies referenced / cited within Acquired documents<br>Current or Draft policy<br>Related to Noise<br>Related to pedestrian amenity<br>Council Plans within 20km from Melbourne's GPO	
Citation searching	5	Cited references were identified through active reading of the documents identified.	6
Contacts	6	None	
Other methods	7	Local Government Council Plans identified using spatial criterion of 20km from Melbourne's GPO to identify the 23 Local Government Areas. The Council Plans were evaluated using relational qualitative content analysis approach.	
**SEARCH STRATEGIES**			
Full search strategies	8	State and Federal policies identified using PRISMA-S Method criteria and evaluated using a hybrid critique and policy review method with an inquiry driven approach. Local Government Council Plans identified using a spatial criterion of LGA within a 20km radius of Melbourne's GPO and evaluated using relational qualitative content analysis approach.	
Limits and restrictions	9	This paper is limited to the needs of the subject matter, and to identify the decision-making frameworks that could be applied to answer the research question. Additionally, the study does not provide a comprehensive understanding of acoustics, data collection methods, pedestrian experiences, or explore the intent, extent, or effectiveness of the policies and approaches beyond the strategies and guidelines related to noise or pedestrian amenities within the urban areas of Melbourne, Australia. Noise policies and provisions focused on aircraft, windfarms, live music, animals, industrial and commercial land uses were excluded from the study as their impacts were not deemed significant from the pedestrian perspective or related to traffic noise.	
Search filters	10	Duplicate documents,<br>Superseded documents,<br>Unrelated to noise pollution,<br>Unrelated to pedestrian amenity,<br>Not specific to Melbourne,<br>Not specific for urban context,	

		Local Government Council Plans beyond 20km of GPO.	
Prior work	11	None	
Updates	12	None	
Dates of searches	13	Council Plans: 19-20 April, 2024, State and Federal Policies: 3-6 June, 2024	
**PEER REVIEW**			
Peer review	14	None	
**MANAGING RECORDS**			
Total Records	15	53	
Deduplication	16	Duplicate documents were sorted manually or not access when identified in other databases.	

PRISMA-S: An Extension to the PRISMA Statement for Reporting Literature Searches in Systematic Reviews
Rethlefsen ML, Kirtley S, Waffenschmidt S, Ayala AP, Moher D, Page MJ, Koffel JB, PRISMA-S Group.
Last updated February 27, 2020.
