# Supplementary material for: The Governance of Traffic Noise Impacting Pedestrian Amenities in Melbourne Australia: A Critical Policy Review
Source: Int J Environ Res Public Health. 2024 Aug 16;21(8):1080. doi: 10.3390/ijerph21081080 (PMC11354694; doi:10.3390/ijerph21081080)
Supplement: Supplementary file 1 [file ijerph-21-01080-s001.zip › ijerph-3074386-supplementary/Table S2 Evaluation of eligible documents.pdf]

Evaluation of the eligible policies, research and advocacy documents.

The document contents were evaluated identifying content related to nine themes as illustrated in the table below. Each theme was graded with one of three classifications: Green = is present in the document. Amber = is only referred to in a general way. Red = is not present in document.

Summary Table of the of 53 document evaluation [%]

Topic	Is present in the document	Is referred to in a general way	Is not Present in the document
Focus on noise	34%	9%	57%
Advocates for reduction in Traffic Noise	36%	4%	60%
Advocates for improved amenities	74%	19%	8%
Focus on pedestrian amenities	53%	21%	26%
Focus on residential amenities	81%	15%	4%
Focus on community outcomes	100%	0%	0%
Focus on transport/Traffic	83%	15%	2%
Focus on health	87%	13%	0%
Focus on environment	90%	10%	0%

- Even though the Council plans do not refer to noise pollution, they have a strong focus on community outcomes including health, the environment and transport. This includes pedestrian amenities and encouraging healthier lifestyles as a preventative health initiative.
- All the documents focus on community outcomes, but most are directed to improving residential amenities. Fewer reference pedestrian amenities and only a couple of policies focus on noise impacting pedestrian amenities.
- The Movement and Place Framework, Plan Melbourne and The Blueprint for Active Australia provide extensive and explicit strategic justification to reduce traffic noise with the aim to protect pedestrian amenities. Of equal significance is the Public Health and Wellbeing Act that is the strategic tool to activate a response to environmental noise nuisances that have impacts on community health. These documents advocate for a reduction in traffic noise, or improved pedestrian amenities.
- Several documents highlight the health, environment and transport benefits attributed to more people walking.

Document Title	Policy type	Author	Author Role	Focus on Noise [Outputs]	[Explicit] Advocates reduction in Traffic Noise	[Implicit] Advocates improved amenities	Focus on pedestrian amenities	Focus on residential amenities	Focus on community [Outcomes]	Focus on Transport / traffic	Focus on Health	Focus on Environment
What Australia Wants 2020 [Victoria]	Survey	The Heart Foundation	Advocacy/ Research									
Blueprint for an Active Australia 2019	Strategy	The Heart Foundation	Advocacy/ Research									
The health effects of environmental noise 2020	Guidelines	enHealth	Advocacy/ Research									
Environmental Noise Guidelines for the European Region 2018	Guidelines	WHO	Advocacy/ Research									
European Union Environmental Noise Directive 2002	Standards	European Union	European Government									
CNOSSOS-EU 2012	Framework	European Union	European Government									
National Environment Protection Measure 2024	Act	Australian Government	Federal Government									
National Environment Protection Council Act 1994	Act	Australian Government	Federal Government									
Road Vehicle Standards Act, 2018	Act	Australian Government	Federal Government									
Vehicle Standard (Australian Design Rule 83/00 – External Noise) 2005	Standards	Australian Government	Federal Government									
National Construction Code 2022	Provisions	Australian Building Codes Board	Federal Government									
Our Cities, Our Future 2011	Policy	Australian Government	Federal Government									
Draft National Urban Policy 2024	Policy	Australian Government	Federal Government									
National Austroads research report 2020	Report	AustRoads	Federal Government									
Banyule Council-Plan-2021-2025	Strategy	Banyule Council	Local Government									
Bayside City Council Plan 2021–2025	Strategy	Bayside Council	Local Government									
Boroondara Community Plan 2021-2031	Strategy	Boroondara Council	Local Government									
Together We Are Brimbank - Council Plan 2021-2025	Strategy	Brimbank Council	Local Government									
City of Darebin Council Plan 2021—2025	Strategy	Darebin Council	Local Government									
Glen Eira COUNCIL PLAN 2021–2025	Strategy	Glen Eira Council	Local Government									
Hobsons Bay COUNCIL PLAN 2021–2025	Strategy	Hobsons Bay Council	Local Government									
Hume-City-Council-Plan-2021-2025	Strategy	Hume City Council	Local Government									
Kingston OUR ROADMAP - Council Plan 2021-25	Strategy	Kingston Council	Local Government									
Manningham Council Plan 2021-2025	Strategy	Manningham Council	Local Government									
Maribyrnong Council-Plan-2021-2025	Strategy	Maribyrnong Council	Local Government									
CITY OF MELBOURNE - CITY OF POSSIBILITY COUNCIL PLAN 2021–2025	Strategy	Melbourne Council	Local Government									

Melton Council-and-Wellbeing-Plan-2021-2025	Strategy	Melton Council	Local Government									
Merribek - [Moreland] Council-Plan-2021-2025	Strategy	Merribek Council	Local Government									
Monash COUNCIL PLAN 2021-2025	Strategy	Monash Council	Local Government									
Mooney Valley Community Vision, Council Plan and Health Plan 2021-2025	Strategy	Mooney Valley Council	Local Government									
Nillumbik-Shire-Council-Plan-2021-2025	Strategy	Nillumbik Council	Local Government									
City of Port Phillip Council Plan 2021-31	Strategy	Port Phillip Council	Local Government									
Future-Stonnington 2021	Strategy	Stonnington Council	Local Government									
WHITEHORSE CITY Council Plan 2021–2025	Strategy	Whitehorse Council	Local Government									
Whittlesea Community Plan 2021-2025	Strategy	Whittlesea Council	Local Government									
Wyndham City Council Plan 2021-25	Strategy	Wyndham Council	Local Government									
Yarra City Council Plan 2021-25	Strategy	Yarra Council	Local Government									
Annoyed by Noise 2021	Guidelines	EPA Victoria	State Government									
Movement and Place in Victoria 2019	Framework	VicRoads	State Government									
Noise Limit and assessment protocol - 1826.4, 2021	Protocols	EPA Victoria	State Government									
Guide to the Environment Protection Regulations – 1753.2, 2021	Framework	EPA Victoria	State Government									
Charter for Human Rights and Responsibilities Act 2006	Act	Victorian Government	State Government									
Public Health and Wellbeing Act 2008	Act	Victorian Government	State Government									
Environment Protection Act 2017	Act	Victorian Government	State Government									
Environment Reference Standard, 2021	Guidelines	Victorian Government	State Government									
Victorian Pedestrian Access Strategy 2010	Strategy	Transport Victoria	State Government									
Safe Pedestrian Program 2023	Program	Transport Victoria	State Government									
Climate Change Act 2017	Act	Victorian Government	State Government									
Noise Control Guidelines 2021	Guidelines	EPA Victoria	State Government									
Transport Integration Act 2010	Act	Victorian Government	State Government									
Plan Melbourne	Strategy	Victorian Government	State Government									
Planning and Environment Act 1987	Provisions	Victorian Government	State Government									
Apartment Design Guidelines for Victoria 2016	Regulations	Victorian Government	State Government									
